# Histological Analysis of Bone Callus in Delayed Union Model Fracture Healing Stimulated with Pulsed Electromagnetic Fields (PEMF)

**DOI:** 10.1155/2021/4791172

**Published:** 2021-08-25

**Authors:** U. Umiatin, Ismail Hadisoebroto Dilogo, Puji Sari, Sastra Kusuma Wijaya

**Affiliations:** ^1^Department of Physics, Faculty of Mathematics and Natural Science, State University of Jakarta, Jakarta, Indonesia; ^2^Doctoral Program in Biomedical Science, Faculty of Medicine, University of Indonesia, Jakarta, Indonesia; ^3^Department of Orthopedic and Traumatology, Faculty of Medicine, University of Indonesia, Jakarta, Indonesia; ^4^Department of Biology, Faculty of Medicine, University of Indonesia, Jakarta, Indonesia; ^5^Department of Physics, Faculty of Mathematics and Natural Science, University of Indonesia, Depok, Indonesia

## Abstract

Delayed union and nonunion fractures are clinical challenges for orthopedic surgeons. The development of fracture complications, such as delayed union and nonunion fractures, is still difficult to predict. Various methods are being investigated to improve fracture healing and prevent complications in patients. There are various methods to promote fracture healing, broadly divided into biological, chemical, and physical methods. One of the most widely used physical methods to promote fracture healing is the pulsed electromagnetic field (PEMF). This study aimed to evaluate the healing process of delayed union fracture after being stimulated by PEMF. Twenty-four rats were randomly divided into two groups: the control group (*n* = 12) and the PEMF group (*n* = 12). Delayed union fracture was performed on the left femur of all rats. Subsequently, the PEMF group was given PEMF stimulus with a magnetic field intensity of 1.6 mT and a frequency of 50 Hz for 4 hours/day and 7 days/week. The fracture healing process was evaluated on days 5, 10, 18, and 28 based on the bone callus histology using safranin O fast green (SOFG) staining. The results of the histological analysis showed that bone cartilage was higher in the PEMF group than in the control group throughout the observation period. In addition, the PEMF group had less fibrous tissue at the beginning of the healing. This finding indicates PEMF stimulation has an effect on inducing osteogenesis on fracture healing and reducing the risk of delayed union.

## 1. Introduction

The prevalence of fracture complications of the long bones, such as delayed union and nonunion fractures, accounts for about 5–10% of all fractures. The Food and Drug Administration (FDA) defines a nonunion as a fracture that does not heal completely after nine months after trauma. Subsequently, a delayed union fracture is defined as the absence of healing observed on radiographic analysis three months after trauma. Nonunion and delayed union are multifactorial complications caused by inadequate reduction, soft tissue damage, infection, fracture fragment disturbance, and poor blood supply. Additionally, diabetes and other systemic diseases also affect the occurrence of fracture healing disorders [1, 2]. Management of nonunion fractures is complex and expensive because of long treatment duration, advanced surgical interventions, and psychological and economic burdens [3].

In recent years, methods to promote delayed union fracture healing with improved efficacy and minimal side effects have been developed, including physical methods. Pulsed electromagnetic field (PEMF) is a biophysical method that the FDA has approved to promote fracture healing. However, the efficacy of this method depends on physical parameters, including frequency, amplitude, and exposure time [4]. The application of PEMF stimulus to promote fracture healing is based on Wolff's law, namely, when given mechanical force, the bone will adapt by deposition of the new bone in the area of compression and absorption of bone in the tension area [5]. PEMF is a method based on indirect electrical stimulation (inductive).

Preclinical studies indicate a relationship between PEMF stimuli and biological responses, including increased proliferation, synthesis and release of growth factors, and bone mineralization rate. PEMF reportedly accelerates osteogenic differentiation of BMSCs culture and increases bone repair, neovascularization, and cell growth in bone necrotic areas. Low frequency (3–3000 Hz) PEMF reportedly increases bone callus mechanical strength and reduces healing time. Zhang et al. reported that exposure to PEMF at a frequency of 50 Hz and magnetic field intensity of 0.8 mT could induce Ca^2+^ release in osteoblasts. Although many studies have shown PEMF stimuli can promote fracture healing, the mechanism of action of PEMF at the cellular and molecular levels is not well understood [6]. It is suspected that PEMF stimulus triggers a biological response through the regulation of specific intracellular pathways [7]. This may be a reason why orthopedic surgeons do not prefer to use the PEMF method. Furthermore, research conducted previously used many variations in physical parameters, leading to inconsistent results [8].

In this study, the effect of PEMF stimulus on fracture healing on the delayed union model in mice will be evaluated. Fracture healing was analyzed based on histological parameters of bone callus using safranin O fast green (SOFG) staining.

## 2. Methods

### 2.1. Study Design

In this study, 24 male Sprague Dawley rats (mean age 12 weeks, mean weight 300 ± 20 g) were used. This study was conducted in the animal laboratory of the Biomedical Research and Development Center. All animals were adapted to laboratory conditions for one week before the experiment. Rats were placed in a plastic cage with three rats per cage. Stable conditions were maintained at ambient temperatures (23 ± 3)°C, stable humidity, and a natural day/night cycle. All rats had access to standard feed and drink. After the delayed union fracture surgery, the rats were randomly assigned to two groups: (1) control group (*n* = 12) and (2) PEMF group (*n* = 12). These two groups were then divided into four subgroups: the 5th, 10th, 18th, and 28th day subgroups. The Ethical Committee of the Faculty of Medicine Universitas Indonesia approved all procedures performed on animals, with approval number 17-05- 0489.

### 2.2. Delayed Union Fracture Model

In this study, surgery was performed to create fractures in the femur of rats. The rats were previously anesthetized using intraperitoneal injection of ketamine (70 milligrams/kg of body weight) and xylazine (35 mg/kg of body weight). Aseptic and antiseptic procedures were performed on the left thigh. A further 2 cm long incision was made on the posterolateral side of the left femur. The fracture area was stripped off the periosteum as long as 5 mm on each side of the fracture in order to produce delayed union [9]. To stabilize the fracture, a 1.2 mm diameter K-wire was inserted into the femur retrogradely through the knee joint. After the fracture procedure, the soft tissues (fascia, subcutaneous, and skin) were sutured using absorbable threads. Subsequently, every rat received intramuscular doses of penicillin and streptomycin (0.1 ml/kg of body weight) and acetaminophen (15 mg/kg of body weight). After surgery, daily observations were conducted on the fracture area to observe for infections.

### 2.3. PEMF Stimulation

**Figure 1:**
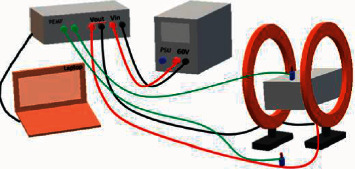
PEMF device representation scheme consisting of Helmholtz coil, pulse generator, and user interface. The PEMF waveform used in experiments consisted of a pulsed burst (burst cycle: 50%, duty cycle: 90%, frequency: 50 Hz). The peak magnetic field intensity within the Helmholtz coil was measured to be approximately 1.6 mT. The cage was placed between the Helmholtz coil.

After fracture surgery and anesthesia recovery, 12 rats in the PEMF group that were divided into four subgroups, each consisting of three mice on the 5th, 10th, 18th, and 28th day, were given the PEMF stimulus. The control group did not receive the PEMF stimulus. The PEMF physical stimulus that was given included a magnetic field intensity of 1.6 mT, frequency of 50 Hz, and stimulus duration of 4 hours/day and 7 days/week. The PEMF device used in this study has been described in previous studies (Figure 1) [10, 11].

### 2.4. Histological Evaluation

During the fracture healing period, on days 5, 10, 18, and 28, three rats from the control and PEMF groups were terminated using lethal doses of ketamine and xylazine. Immediately after the rats died, the left femur was removed for histological analysis. After the left femur was cleaned of soft tissue, the bone was fixed in 4% paraformaldehyde (PFA) for 24 hours, followed by decalcification using hydrochloric acid (Shandon TM TBD-1TM Rapid Decalcifier, Thermo Scientific). After decalcification was complete, the specimens were embedded in paraffin and cut longitudinally to a thickness of 5 *μ*m. Samples were then stained using safranin O fast green. The image reconstruction was observed using a Nikon Eclipse optic microscope. The percentage value of cartilage and bone was determined using ImageJ software (National Institutes of Health, USA) [12].

## 3. Results

### 3.1. Mortality Rate

Fracture healing involves complex interactions between anatomical, biomechanical, and biochemical processes. In this study, a fracture was made on the delayed union model by stripping the periosteum of the left femur in rats. At the end of the study, no rats died, and every rat showed normal activity (Table 1).

### 3.2. Histological Evaluation

**Figure 2:**
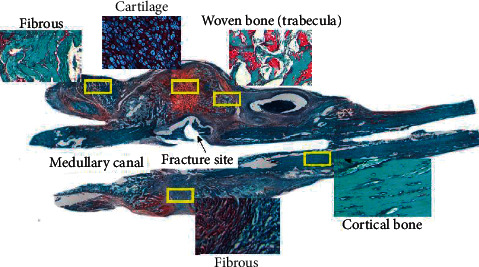
Microscopic images of longitudinal sections of the femur were stained with safranin O fast green (SOFG) on day 10. Bone callus consisted of fibrous, cartilage, and bone tissue. Using SOFG staining, cartilage tissue is observed as reddish-orange, while other connective tissues are observed as green. Longitudinal sections of bone at 40x magnification and several sections of bone at 200x magnification were observed (yellow square line).

The healing process was analyzed histologically on days 5, 10, 18, and 28 postfracture. Accurate histological measurement of bone callus was essential to validate PEMF stimulus efficacy in fracture healing. Histological images provide information on important stages of the healing process based on cellular and tissue responses. Representative microscopic longitudinal sections of the femur were shown in Figure 2.

**Figure 3:**
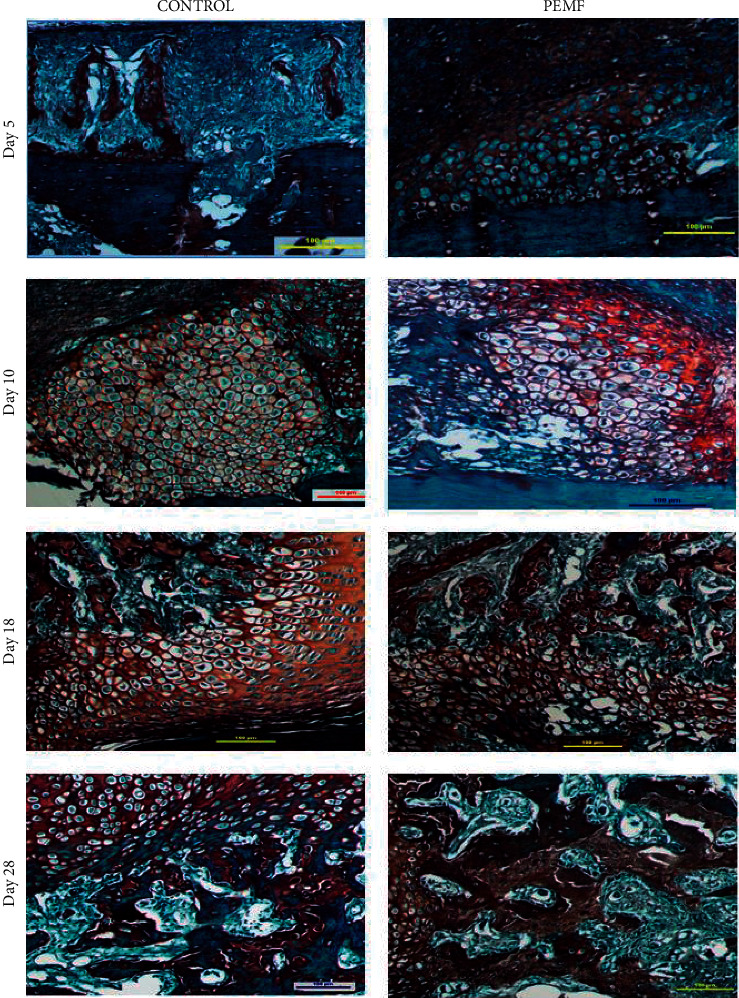
Comparison images of bone callus histology between the control and PEMF groups on days 5, 10, 18, and 28 after fracture. SOFG staining shows cartilage as orange and bone as blue. The PEMF group on day 5 showed less fibrous tissue than the control group. Image magnification ×100. Scale bar: 1 mm.

At each of these stages, the histological features of bone callus stained with SOFG were observed and compared between the control and PEMF treatment groups (Figure 3). SOFG staining indicates cartilage (orange) and bone tissue (blue).

**Figure 4:**
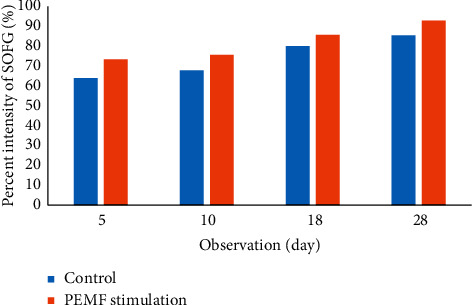
Percent intensity of SOFG-stained in control and PEMF groups on days 5, 10, 18, and 28.

The percent intensity of bone-cartilage stained by SOFG gradually increased from day 5 to day 28 (Figure 4). The percent intensity of control group was 63.87%, 67.76%, 80.01%, and 85.32% on days 5, 10, 18, and 28, respectively, while PEMF group was 73.27%, 75.50%, 85.53%, and 92.76% on days 5, 10, 18, and 28.

## 4. Discussion

Pulsed electromagnetic field stimulus (PEMF) is an inductive electrical stimulus using a dynamic magnetic field to produce an induced electric field. This method is widely used to stimulate peripheral and central nerves. PEMF has also been reported to positively affect the repair, homeostasis, and bone remodeling process [13].

The histology of fracture healing consists of four overlapping phases, starting with an inflammatory response, soft callus formation consisting of fibrous tissue and cartilage, formation of hard callus (woven bone), and remodeling. Fracture healing in the diaphysis area occurs through endochondral and intramembranous ossification. Endochondral ossification is the process of bone formation during the cartilage phase, characterized by hypertrophy, calcified chondrocytes in the cartilage matrix followed by angiogenesis. Thereafter, osteoblasts will synthesize woven bone (immature bone) in the collagen tissue produced by chondrocytes. Evaluation of the cartilage composition of the callus promptly can be a sensitive index of the fracture healing progress. Delays in chondrogenesis and cartilage calcification have been reported to indicate delayed union and nonunion in several clinical and in vivo studies [14].

In this study, fracture healing was assessed by comparing the characteristics of fibrous tissue and cartilage in the callus control group and the PEMF exposure group. When HE staining is used, it is sometimes difficult to distinguish tissue morphology and quantify it. Therefore, a special safranin O fast green (SOFG) stain was used. The presence of type II collagen characterizes cartilage tissue, an abundance of glycosaminoglycan (GAG), and the absence of type I collagen. Glycosaminoglycan is negatively charged, so cationic staining with SOFG is appropriate [15].

Histological evaluation was conducted on day 5, where healing begins to occur, marked by the formation of a hematoma to isolate the damaged tissue. During this phase, granulation tissue was formed in both the control and PEMF groups in the fracture area. Granulation tissue is a primitive cell source that contains many growth factors. The initial stage of fracture healing is also characterized by the formation of fibrous tissue in the fracture area. Fibrous tissue consists mainly of fibroblasts and a collagen matrix and plays a role in oxygen and nutrient transport throughout the body, as a link between bones and protects muscles from injury. Fibrous tissue, also termed as dense connective tissue, consists of collagen arranged irregularly [1]. In the PEMF group, less hyaline cartilage tissue in the proliferation phase was found; however, this was still more than in the control group. In the control group, the fracture area still consisted of a copious amount of fibrous tissue. Abundant fibrous tissue in the intramembranous and endochondral ossification phase indicates delayed healing and nonunion potential [14].

On the 10th day after fracture, scant fibrous tissue was still visible in the fracture area, but hypertrophied chondrocytes began to appear. Day 10 is part of the reparative phase of fracture healing and is characterized by the formation of a soft callus composed of fibrous and hyaline cartilage. In this phase, the process of intramembranous and endochondral ossification occurs [16]. An orange cartilage matrix was observed. The PEMF group showed an increase in bone matrix production compared to the control group. On the 10th day of observation, there was a significant reduction in the fibrous area of the PEMF exposure group. The process of resorption of fibrous tissue and cartilage formation simultaneously affects bone tissue formation in the next phase.

On day 18, cartilage was transformed into woven bone; hypertrophied cartilage began to calcify into woven bone. Woven bones dominated the callus. Woven bone is immature bone characterized by random collagen orientation without a Haversian system. Osteoblast-like cells are seen in rows on the woven bone. On the 18th day, a small amount of cartilage in the hypertrophy phase was still seen both in the control and PEMF exposure groups, indicating that the endochondral ossification phase had not yet ended. This phase is also characterized by proteoglycan loss from the matrix around chondrocytes. These results are consistent with molecular examinations of *β*-catenin expression conducted in previous studies. The highest *β*-catenin expression occurred on day 18 after fracture [17]. The expression of *β*-catenin in hypertrophic chondrocytes encourages the expression of osteopontin, which is the primary matrix protein in cartilage and bone [18, 19].

Bone formation is preceded by the deposition of an irregularly oriented bone matrix known as woven bone or trabecular bone. In the final stage of fracture healing, the trabecular bone undergoes remodeling to form cortical bone (lamellar bone) through a series of osteoblast and osteoclast activities. Cortical bone is characterized by a structured collagen orientation and the presence of a Haversian system [20]. The rate of cartilage resorption strongly influences the rate of bone remodeling [21].

Observation on day 28 represents the initial phase of remodeling. Based on histological observations on day 28, the callus of both control and PEMF groups was dominated by woven bone. A similar observation was made by Beil et al., who examined fracture healing in rats [22]. These results are consistent with molecular examination results in previous studies. The results of qRT-PCR analysis showed that the expression of Wnt5a, Wnt10b, and *β*-catenin was higher in the PEMF group than the control group. These genes are related to the activity of osteoblast in synthesizing the bone matrix. Moreover, on the 28^th^ day, decreased *β*-catenin expression was observed. A decrease in *β*-catenin expression plays a role in encouraging preosteoclasts differentiation into mature osteoclasts that will absorb bone [17]. In previous studies, based on molecular analysis, an increase in the expression of RANK, RANKL, and OPG in the PEMF group was observed. These transcription factors are related to the regulation of osteoblast and osteoclast activity during bone formation [23].

Overall, this study shows that PEMF accelerated bone formation in the early phase of fracture healing, as indicated by a decrease in the fibrous tissue area and an increase in the cartilage area in the PEMF group. These results are consistent with previous studies, which showed a significant increase in the alkaline phosphatase (ALP) enzyme on day 10. ALP is an enzyme that affects osteoblast activity in synthesizing bone matrix. Until now, the molecular mechanism underlying PEMF fracture healing promotion is still being investigated. A signaling pathway may be involved in the Wnt signaling pathway [17].

In this study, the longitudinal cutting technique is a study weakness. The advantage of a longitudinal cut is that it can provide a complete picture of the callus by combining a series of histological images. However, the disadvantage is that maximum magnification cannot be achieved when taking histological images.

## 5. Conclusion

Based on the results of this study, PEMF could potentially increase osteogenesis in fracture healing, evidenced by a small amount of fibrous tissue and a high amount of cartilage in the initial healing phase. These results indicate PEMF has a more positive effect when utilized at the early stage of fracture healing.

## Figures and Tables

**Table 1:** Observation of rats' mortality.

Percent survival (%)	Observation (day)			
	5	10	18	28
Control	100	100	100	100
PEMF stimulation	100	100	100	100

## Data Availability

All relevant data are included within the article.
